# Draft Genome Sequences of Two O104:H21 *Escherichia coli* Isolates Causing Hemorrhagic Colitis during a 1994 Montana Outbreak Provide Insight into Their Pathogenicity

**DOI:** 10.1128/genomeA.00805-13

**Published:** 2013-10-03

**Authors:** Narjol Gonzalez-Escalona, Melinda A. McFarland, Lydia V. Rump, Justin Payne, Denis Andrzejewski, Eric W. Brown, Peter S. Evans, Timothy R. Croley

**Affiliations:** Division of Microbiology, Center for Food Safety and Applied Nutrition, Food and Drug Administration, College Park, Maryland, USAa; Division of Analytical Chemistry, Center for Food Safety and Applied Nutrition, Food and Drug Administration, College Park, Maryland, USAb; Department of Nutrition & Food Science, University of Maryland, College Park, Maryland, USAc

## Abstract

We sequenced the genomes of two strains of O104:H21 enterohemorrhagic *Escherichia coli* (EHEC) isolated during an outbreak of hemorrhagic colitis in Montana in 1994. These strains carried a plasmid that contains several virulence genes not present in pO157. The genome sequences will improve phylogenetic analysis of other non-O157 *E. coli* strains in the future.

## GENOME ANNOUNCEMENT

Enterohemorrhagic *Escherichia coli* (EHEC) serotype O157:H7 causes most of the world’s food-borne outbreaks of *E. coli* infection (1), but outbreaks caused by non-O157 EHEC strains have increased (1) and now account for 20 to 50% of all U.S. Shiga toxin-producing *Escherichia coli* (STEC) illnesses (2). A 1994 Montana outbreak was caused by *E. coli* O104:H21, which produced Shiga toxin 2 and carried *hlyA* (*ehxA*) but was negative for the intimin gene (3). The recent *E. coli* O104:H4 outbreak in Germany (4), considered the largest outbreak of non-O157 *E. coli* illness recorded to date, has renewed interest in these important non-O157 pathogens. We sequenced the genomes of two O104:H21 strains involved in the Montana outbreak: ATCC BAA-178 and ATCC BAA-182.

DNA from each strain was isolated from overnight cultures using the DNeasy blood and tissue kit (Qiagen, Valencia, CA). The genomes were sequenced using the Ion Torrent (PGM) sequencing system with 200-bp read chemistry (Life Technologies) at 30× coverage. The Ion PGM 200 sequencing kit was used according to the manufacturer’s instructions. Genomic sequence contigs for each strain were *de novo* assembled using the CLC Genomics Workbench, version 5.5.1 (CLC bio, Germantown, MD). The G+C content for both strains was 50.6 mol%—similar to the G+C contents for other *E. coli* strains. ATCC BAA-182 has 198 contigs, ranging from 533 to 223,254 bp, with a total size of 4,929,288 bp. ATCC BAA-178 has 175 contigs, ranging from 540 to 273,952 bp, with a total size of 4,940,080 bp.

The draft genome sequences were annotated using the NCBI Prokaryotic Genomes Automatic Annotation Pipeline (PGAAP) (http://www.ncbi.nlm.nih.gov/genomes/static/Pipeline.html). Strains were identified as sequence type 672 (ST-672) by *in silico* multilocus sequence typing (MLST) (Center for Genomic Epidemiology; http://cge.cbs.dtu.dk/services/) (5) using the MLST database *E. coli* no. 1 (http://mlst.ucc.ie/mlst/dbs/Ecoli) and as an unknown ST using the MLST database *E. coli* no. 2 (http://www.pasteur.fr/recherche/genopole/PF8/mlst/EColi.html). Both were identified as ST-50 (15 genes) and ST-123 (7 genes) using the *E. coli* MLST database (http://www.shigatox.net/ecmlst/cgi-bin/scheme).

O104/Ount:H21 *E. coli* strains 02-03885 and 3356/97/B from ST-672 cause hemolytic-uremic syndrome (HUS) but also carry the *stx*_1_ and *stx*_2d_ genes (6). No antibiotic resistance genes were detected by *in silico* screening (7). This approach does not detect mutations conferring chromosomal antibiotic resistance (i.e., to nalidixic acid [NAL]) (7). When we screened against 16 different variants of 9 known intimins (8), no intimin gene (*eae*) was found, which was consistent with previous reports of these strains being negative by PCR for *eae*-γ and *eae*-β (3). Eight prophage regions were detected using PHAST. One of the incomplete prophages contained the *stx*_2_ genes, suggesting that it is a new and previously undescribed phage.

Both strains also carried a plasmid highly similar in organization and content to pO113, present in an *E. coli* O113:H21 strain. This plasmid carried several virulence genes, including *epeA* (encoding autotransporter EpeA, an extra serine protease), *saa* (encoding autoagglutinating adhesion Saa), and subtilase cytotoxin genes (*subAB*), which are not found on pO157 carried by O157:H7 (9, 10). Possession of this pO113-like plasmid plus the H21 flagellin might explain the virulence of the O104:H21 *eae-*negative strains, which resembles the virulence of *eae-*negative *E. coli* O113:H21 strain 98NK2 (10).

A detailed report on the phylogenetic analysis of the draft genome sequences will be included in a future publication.

### Nucleotide sequence accession numbers.

The draft genome sequences of the two *E. coli* strains are available in GenBank under accession numbers AUQB00000000 for strain ATCC BAA-182 (CFSAN002237) and AUQC00000000 for strain ATCC BAA-178 (CFSAN002236).
